# 3D Defect Localization on Exothermic Faults within Multi-Layered Structures Using Lock-In Thermography: An Experimental and Numerical Approach

**DOI:** 10.3390/s17102331

**Published:** 2017-10-13

**Authors:** Ji Yong Bae, Kye-Sung Lee, Hwan Hur, Ki-Hwan Nam, Suk-Ju Hong, Ah-Yeong Lee, Ki Soo Chang, Geon-Hee Kim, Ghiseok Kim

**Affiliations:** 1 Optical Instrumentation Development Team, Korea Basic Science Institute, 169-148 Gwahak-ro, Yuseong-gu, Daejeon 34133, Korea; baejy@kbsi.re.kr (J.Y.B.); kslee24@kbsi.re.kr (K.-S.L.); hurhwan@kbsi.re.kr (H.H.); namkihwan@kbsi.re.kr (K.-H.N.); ksc@kbsi.re.kr (K.S.C.); kgh@kbsi.re.kr (G.-H.K.); 2 Department of Biosystems and Biomaterials Science and Engineering, Seoul National University, 1 Gwanak-ro, Gwanak-gu, Seoul 08826, Korea; hsj5596@snu.ac.kr (S.-J.H.); ayoung3308@snu.ac.kr (A.-Y.L.)

**Keywords:** infrared thermal microscopy, lock-in method, nondestructive test, finite element simulation, silicon wafer stacked heat source chip

## Abstract

Micro-electronic devices are increasingly incorporating miniature multi-layered integrated architectures. However, the localization of faults in three-dimensional structure remains challenging. This study involved the experimental and numerical estimation of the depth of a thermally active heating source buried in multi-layered silicon wafer architecture by using both phase information from an infrared microscopy and finite element simulation. Infrared images were acquired and real-time processed by a lock-in method. It is well known that the lock-in method can increasingly improve detection performance by enhancing the spatial and thermal resolution of measurements. Operational principle of the lock-in method is discussed, and it is represented that phase shift of the thermal emission from a silicon wafer stacked heat source chip (SSHSC) specimen can provide good metrics for the depth of the heat source buried in SSHSCs. Depth was also estimated by analyzing the transient thermal responses using the coupled electro-thermal simulations. Furthermore, the effects of the volumetric heat source configuration mimicking the 3D through silicon via integration package were investigated. Both the infrared microscopic imaging with the lock-in method and FE simulation were potentially useful for 3D isolation of exothermic faults and their depth estimation for multi-layered structures, especially in packaged semiconductors.

## 1. Introduction

The miniaturization of electronic devices has led to the advancement of three-dimensional (3D) integration technologies, which are a fabrication method in which dices or wafers are stacked in the height direction and then interconnected perpendicularly and/or horizontally. These technologies support the following features: multifunction, smaller form factor, higher integration density, more advanced electrical characteristics and reliability, higher communication bandwidth, less communication power, less production cost, and effective thermal dissipation [1]. However, this trend has also resulted in a new increased structural complexity of microelectronic devices. Also, new types of failures arising in service or fabrication process can occur. Consequently, fault isolation (FI) or failure analysis (FA) tasks for the identification of defects in the integrated circuits (ICs) become more complicated.

Because local exothermic faults, one of the major types of faults possible in microelectronic devices, may be caused by electrical short circuits, junction breakdowns, high resistive opens, or other kinds of internal faults [2,3], microscopic thermography methods can be a very useful tool for FI or FA. Several thermography techniques for microelectronic devices have been developed and utilized to analyze the thermal characteristics of them, such as photon emission microscopy (PEM) [4], liquid crystal thermography (LCT) [5], fluorescence micro-thermography (FMT) [6], scanning thermal microscopy (SThM) [7], steady-state infrared thermography (IRT) [8], and thermo-reflectance microscopy (TRM) [9]. However, these techniques are not applicable to locate the defects at interconnect levels in miniature multi-layered integrated architectures such as microelectronic devices, because they have some limitations in the depth estimation of heat sources which are buried in multi-layered opaque materials. To overcome this limitation, lock-in thermography (LIT) has been developed by incorporating a lock-in algorithm into the infrared thermal sensing technique [10,11,12,13,14]. LIT enables the effective spatial and temperature resolutions to be improved through the dynamic and averaging nature of it. Therefore, it facilitates the detection of exothermic fault by providing enhanced position or depth information of fault [15].

Since the seminal work on LIT by Busse et al. [10], many research efforts have been devoted to the application of LIT to ICs, semiconductor packages, or semiconductor devices for FA [2,3,12,16,17,18,19]. Breitenstein et al. [12] investigated the shunting and leakage phenomena in electronic devices such as solar cells and metal oxide semiconductor (MOS) structures by developing dynamic precision contact thermography (DPCT), which is the first LIT technique enabling the detection of temperature of 100 μK with a spatial resolution of 30 μm. Rakotoniaina et al. [2] improved the performance of LIT systems to include enhanced temperature and spatial resolution functions, and to non-contact methods, and used this system for detecting local shunts in solar cells and for localizing weak heat sources in ICs. These studies provided valuable data to establish LIT for application to semiconductor packages; however, it is restricted to detecting the 2D failure location only. More recently, Schmidt et al. [3] experimentally showed that LIT can be used for 2D defect localization on decapsulated and fully packaged ICs and for 3D faults isolation in a stacked die device by calculating the phase shift. However, they used a heat source configuration that only passes the covering encapsulation material, such as a mold compound with low thermal diffusivity, to demonstrate the principle of 3D hotspot localization.

Because actual IC or semiconductor packages are stacked by dissimilar materials with different thermal properties, e.g., thermal diffusivity, the influence of these properties has to be considered when calculating the phase shift. Considering these facts, the phase shift for 3D defect localization on the effects of the thermal contact resistance between two different layers should be quantified by various heat source configurations [18]. Kijkanjanapaiboon et al. [19] investigated the accuracy of the interpretation of the LIT data based on semi-infinite models, which have a heat source located at the origin and homogeneous material with infinite dimensionality, by using an analytical solution and finite element (FE) analysis. They focused on analyzing the effects of using finite dimensionality, heat source configuration, the convective heat transfer coefficient, the thickness and thermal diffusivity of the material, and the lock-in frequency in a homogeneous and single material, by calculating the difference in the phase shift and the estimated depth of the error. They also investigated the effect of the shapes and locations of the heat source and the multiple materials, by calculating the phase shift only. Even though these studies addressed the problem relating to the use of multiple materials, they did not present an estimation of the depth of the actual defect within the multi-layered structures. Furthermore, their examination was based on a semi-infinite model only. Actually, to locate the depth using LIT, it is of considerable importance to understand the effect on the 3D defect localization by the system parameters and sample properties such as the sensitivity of the infrared camera, the optical properties of material such as transparency and emissivity, the thermal properties of the material such as thermal diffusivity, the sampling rate, and heat source configuration [19,20]. Therefore, the combination of experimental and numerical demonstrations for actual LIT applications is very important for the success of 3D defect localization in 3D IC or semiconductor packages.

The objective of this study was to demonstrate the feasibility and superiority of LIT method for the 3D localization of an exothermic fault within multi-layered structures through experimental and numerical approaches. For this goal, an infrared microscopic imaging system was constructed, and two silicon wafer stacked heat source chips (SSHSCs) were fabricated. In addition, the 3D FE models of the SSHSCs were developed for the coupled electro-thermal simulation of them. The specific objectives of this study were to analyze phase shifts of transient thermal responses; secondly, to estimate the depth of heat source buried in test samples (SSHSCs) by using LIT experiments and FE simulations; and, to investigate the effects of the volumetric heat source configuration on the depth estimation by carrying out a FE simulation of through silicon via (TSV) mimicking models.

## 2. Lock-In Principle

**Figure 1:**
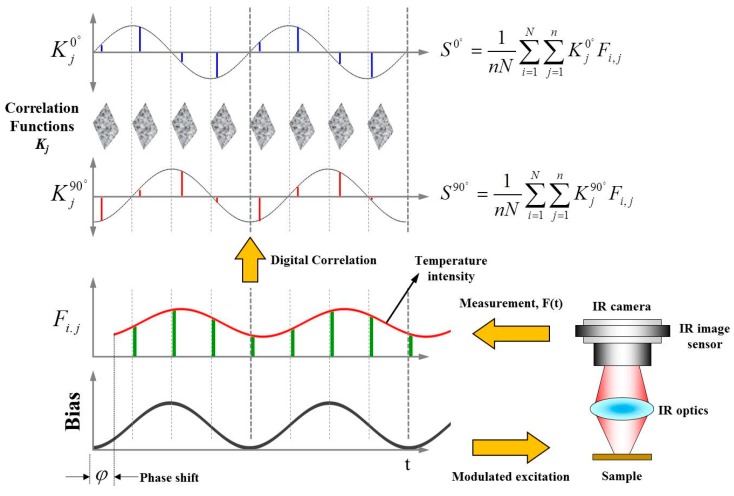
Working principle of an infrared lock-in operation.

Figure 1 shows the working principle of the LIT technique used in this study. Modulated bias applied to the test specimen induces the periodical thermal emissions from its hotspot. The periodical thermal emissions can be changed by the physical and thermal properties of the materials that form the test specimen. In addition, the calculated phases of periodical thermal emissions can be altered by the depth of the hotspot within a test specimen. The periodical thermal images are measured by an infrared thermal camera, and then a modulated wave pattern is reconstructed by selecting thermal images at intervals of *T/n* (where *T* means a lock-in period, and *n* is the sampling number per one period). For the real-time lock-in computing of the periodical thermal emissions, we employed a lock-in correlation operation during measurements. This method has effects of averaging the product of the measured thermal signals with a correlation function during its operations. The general result of lock-in correlation operation can be calculated as the summation *S* (where *N* is a number of period, and *n* is the sampling number per one period) as shown in Equation (1) [20]:

(1) $$S=\frac{1}{nN}\sum_{i=1}^{N}\sum_{j=1}^{n}K_{j}F_{i,j}$$

The lock-in correlation technique uses two channels of correlation functions *K_j_* to reconstruct both sine and cosine components for the measured thermal signals. The measured thermal signals in accordance with a sine curve are calculated by the first channel of correlation function, and the thermal signals in accordance with a cosine function are reconstructed by the second channel of correlation function, which is 90° phase shifted from the first channel of correlation function. If the amplitude and phase of the modulated thermal signals are set to *A* and *ϕ*, the measured thermal signal *F*(*t*) can be defined as *A*sin(2*πf_lock-in_t*)cos*ϕ* + *A*cos(2*πf_lock-in_t*)sin*ϕ* by considering both modulated bias and periodical thermal emissions. Then the signal *F*(*t*) has been processed by correlating the two channels of functions such as *K*^0°^(*t*) *=* 2sin(2*πf_lock-in_t*) and *K*^90°^(*t*) = 2cos(2*πf_lock-in_t*). Both *S*^0°^ = *A*cos(*ϕ*) and *S*^90°^ = *A*sin(*ϕ*) are the result of the digital lock-in correlation method during whole measurements. They are commonly called as the in-phase (*S*^0°^) and the quadrature signal (*S*^90°^). As described in Equations (2) and (3), the phase-independent amplitude *A*, and the phase *ϕ* are numerically calculated from the *S*^0°^ and *S*^90°^ [20]:

(2) $$A=\sqrt{{\left(\;S^{0^{o}}\right)}^{2}+{\left(\;S^{90^{o}}\right)}^{2}}$$

(3) $$\varphi =\arctan\left(\frac{S^{90^{o}}}{S^{0^{o}}}\right)$$

## 3. Experiment

### 3.1. Silicon Wafer Stacked Heat Source Chip

**Figure 2:**
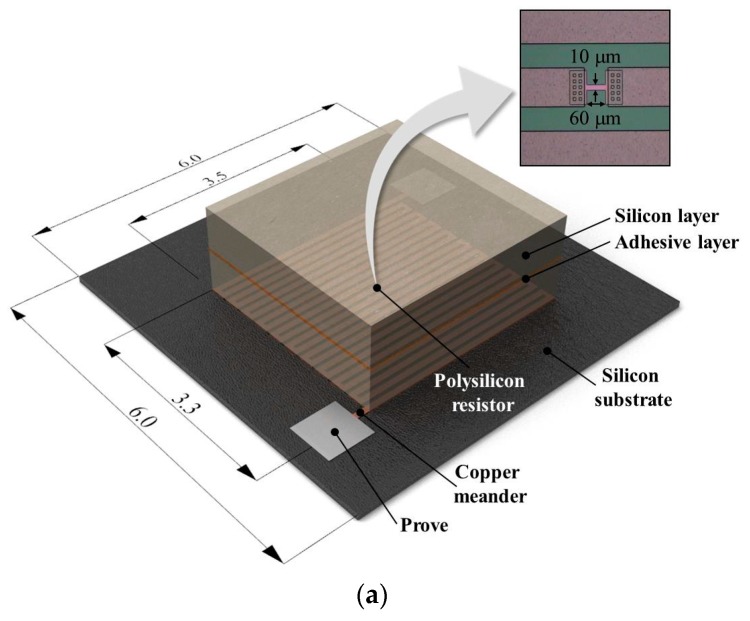

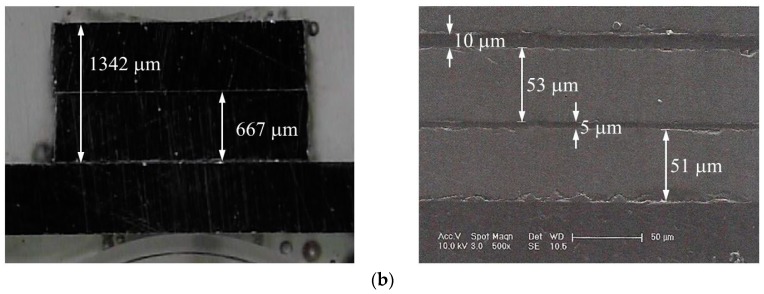
(**a**) Schematic of the silicon wafer stacked heat source chip (SSHSC); (**b**) Cross-section image of the two-layer SSHSC 1342 µm in height (**left**) and three-layer SSHSC 170 µm in height (**right**).

Recently, lots advanced micro-electronic devices have displayed short failures due to shrinking design nodes. Especially D-RAM, FLASH, LSI devices require a way to see short failures. Because short failures always generate a heat (thermal emission) signature due to metal contact, we designed and fabricated two kinds of SSHSCs as an artificial heat sink multi-layered structure sample by considering both the bonding structure of dissimilar materials, and the heat generation by short failure. The SSHSCs comprise two parts: (1) the heat generation part that contains the silicon substrate, polysilicon resistor, and copper meander, and (2) the wafer stacked part that includes multi-layered silicon and adhesive as shown in Figure 2a.

Heat was induced by Joule heating by locally connecting the polysilicon resistor to the central region of the copper meander (Figure 2a). Consequently, periodical biases supplied to the copper meander induce the modulated heat from the position of the polysilicon resistor, and the generated heat will be transferred through the stacked layers. Figure 2b shows cross-sectional images of the two-layer SSHSC 1342 µm in height and three-layer SSHSC 170 µm in height; the total thicknesses of each layer on the silicon wafer and adhesive are 1334 μm and 8 μm for the two-layer SSHSC, and 150 μm and 20 μm for the three-layer SSHSC, respectively.

### 3.2. Lock-In Thermography

**Figure 3:**
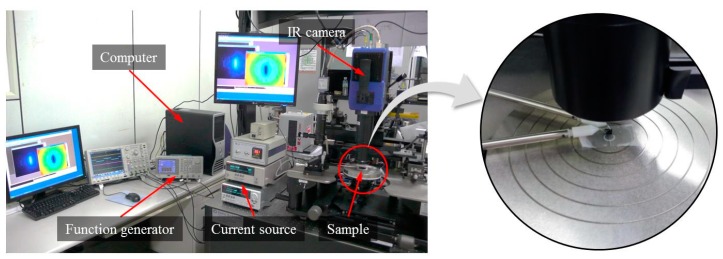
Infrared lock-in microscope system.

Figure 3 shows the infrared microscopic imaging system used in the lock-in operation of thermal emissions. It consists of an infrared camera, infrared microscopic lens, function generator and current source. Pixel resolution, spectral band, and temperature sensitivity of an infrared camera (SC7600, FLIR Systems, Wilsonville, OR, USA) used in study are 640 × 512 pixels, 1.5~5 µm, and 18 mK at 25 °C, respectively.

The thermal imaging detector in the infrared camera was indium antimonide (InSb) and employed an integrated stirling cooler for its stable operation. An infrared microscopic lens with a 3.2 × 2.5 mm field of view and 3.5–5 μm spectral band was embedded in an infrared camera. In addition, a current source (2602B, Keithley Instruments Inc., Cleveland, OH, USA) was used to supply a modulated bias by precisely controlling the frequency, voltage amplitude, and duty rate of the bias signal. The test specimen was tightly positioned using a vacuum chunk during measurement in order to remove any possible vibration noise. For these LIT experiments, increasing lock-in frequencies were chosen to achieve good spatial confinement of the heat around local heat sources. Considering the thickness and configuration of the stacked materials for each test specimen, various lock-in frequencies were selected as 1, 2, 3, 4, and 5 Hz for two-layer SSHSC and as 2, 4, 6, 8, and 10 Hz for three-layer SSHSC, respectively. It is known that the higher the lock-in frequency, the higher the effective spatial resolution. Therefore, we selected two groups of increasing lock-in frequencies to enable us to consider both the spatial resolution and signal-to-noise ratio (SNR) simultaneously. Three modulated bias voltages of 3 V, 4 V, and 5 V were used for the LIT experiments and simulations in this study because modern integrated circuits of TTL and CMOS series are commonly driven by the biases range from 3 V to 5 V. The sampling rate used in this experiment was 33 Hz for all the measurements.

### 3.3. Depth Estimation

LIT basically uses the effect of the time-dependent thermal wave propagation inside the material, which depends on the thermal stimulation frequency, distance between the hotspot and specimen surface, and thermal properties of the material. It means that the time delay caused by the attenuation during the thermal diffusion determines the phase shift (*ϕ*) between the modulated bias signal and periodical thermal emission at the top surface of specimen. The depth (*z*) of the actual defect was estimated by the relationship between the phase shift and thermal diffusion length, expressed as in Equation (4):

(4) $$\varphi =\frac{z}{\mu }\times 180/\pi$$

Here, the parameter, thermal diffusion length (*μ*), which reflects the influence of the physical and thermal properties of test material, shows the thermal wave damping through the material. For a semi-infinite homogeneous material model, the thermal diffusion length (*μ*) is given by [21]:

(5) $$\mu =\sqrt{\frac{2\alpha }{\omega }}=\sqrt{\frac{\alpha }{\pi f_{lock-in}}}$$

where α (m^2^/s) is the thermal diffusivity defined as $\alpha =k/\rho C_{p}$, where k (W/m·K) is the thermal conductivity, $C_{p}$ (J/kg·K) is the specific heat capacity at constant pressure, ρ (kg/m^3^) is the density, and ω (Hz) is the modulation frequency defined as $\omega =2\pi f_{lock-in}$, where $f_{lock-in}$ (Hz) is the applied lock-in frequency.

**Figure 4:**
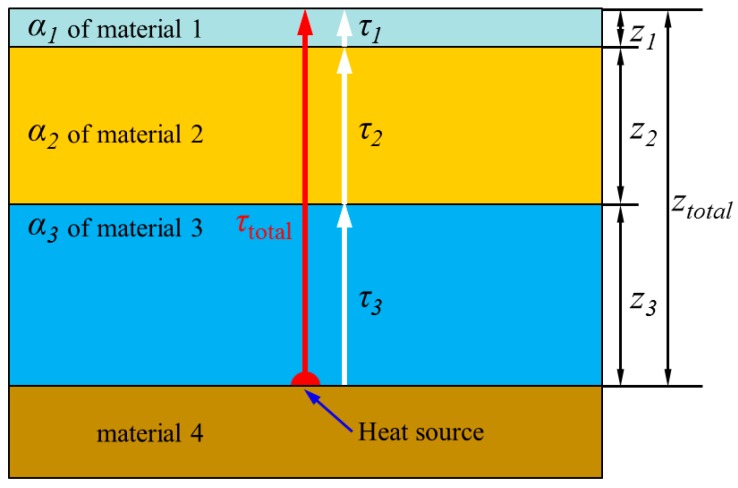
Multi-layered structure stacked by dissimilar materials with different thermal diffusivity.

However, actual 3D IC or semiconductor packages not only have a finite dimension but also multi-layered structures stacked by dissimilar materials with different thermal diffusivity, as shown in Figure 4. Taking into account these facts, we determine the effective thermal diffusion length ($\mu_{eff}$) by considering the calculated time required for heat diffusion to pass through each stacked layer. The thermal diffusivity of the stacked material can be used to calculate the time (τ) of heat propagation for the layer thickness (l) according to following Equation (6) [22]:

(6) $$\alpha =\frac{l^{2}}{\tau },\;\tau =\frac{l^{2}}{\alpha }$$

For the finite dimension and multi-layered structures, the total time ($\tau_{total}$) for heat diffusion to pass through the entire thickness of the stacked layers from heat source ($z_{total}$) is given by:

(7) $$\tau_{total}=\frac{{l_{1}}^{2}}{\alpha_{1}}+\frac{{l_{2}}^{2}}{\alpha_{2}}+\ldots \frac{{l_{n}}^{2}}{\alpha_{n}}=\tau_{1}+\tau_{2}+\ldots \tau_{n},\;\tau_{total}=\sum_{i=1}^{n}\tau_{i}$$

Substituting Equation (7) into Equation (6), we obtain the effective thermal diffusivity ($\alpha_{eff}$), expressed as:

(8) $$\alpha_{eff}=\frac{z_{total}^{2}}{\tau_{total}}$$

Using the effective thermal diffusivity ($\alpha_{eff}$), the effective thermal diffusion length ($\mu_{eff}$) can be determined as:

(9) $$\mu_{eff}=\sqrt{\frac{\alpha_{eff}}{\pi f_{lock-in}}}$$

Thus, estimation of the actual depth of the local heat source ($z_{total}$) in terms of the finite dimension and multi-layered structures can be carried out using the effective thermal diffusion length ($\mu_{eff}$) and the phase information, according to following Equation (10) [3,16].

(10) $$z_{total}=\mu_{eff}\times \varphi \times \pi /180$$

## 4. Numerical Simulations

### 4.1. Finite Element Modeling

**Figure 5:**
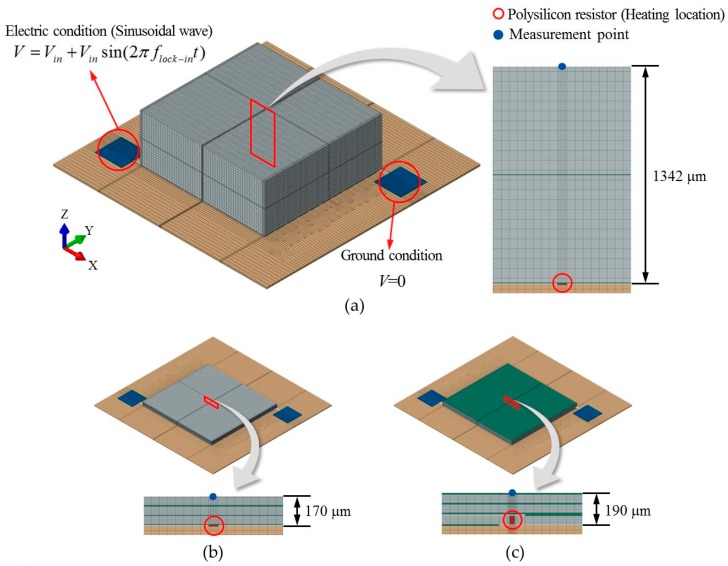
3D FE models used in FE simulation: (**a**) two-layered SSHSC; (**b**) three-layered SSHSC; (**c**) 3D TSV integrated package purposed model.

The thermal responses from the top surface of each test specimen heated by the electric current (known as Joule heating) were analyzed via coupled electro-thermal simulation. Two 3D FE models for the two-layer and three-layer SSHSCs were constructed based on the geometric dimensions of test specimens that were used in an infrared lock-in experiment (see Figure 5a,b). Another FE model for the 3D TSV integrated package was also constructed to investigate the effects of the volumetric heat source configuration (see Figure 5c). These FE models involve electro-thermal behavior which is governed by the Maxwell equation and heat equation. The governing equation in the transient state of nonlinear heat transfer with its general form in Cartesian coordinates, applied to the computational domain, can be written as:

(11) $$\rho C_{p}\frac{\partial T(r,t)}{\partial t}=\nabla \cdot [k\nabla T(\vec{r},t)]+Q_{J}$$

where T (K) is the temperature. Further, $Q_{J}$ (W/m^3^) is the volumetric heat source generated by Joule heating and can be represented by the quantity of ohmic losses, that is, the power per unit volume lost in the form of heat from the electric fields, and is defined as:

(12) $$\frac{dP_{loss}}{d\nu_{vol}}=JΕ=Q_{J}$$

where $P_{loss}$ (W) is the power lost from the electric field, $\nu_{vol}$ (m^3^) is the volume, J (A/m^2^) is the current density, and Ε (V/m) is the electric field. From Ohm’s law and Maxwell’s equations, we obtain:

(13) J=σΕ

(14) Ε=−∇V

(15) $$\frac{\partial q}{\partial t}+\nabla \cdot J=0$$

where σ (S/m) is the electric conductivity, V (volt) is the electric potential, and q (coulomb/m^3^) is the volume charge density, and the electric field can be calculated using the above equations. Solving the aforementioned equations simultaneously enabled the electric current density, electric potential gradient, distributions of the heat flux and temperature, and transient temperature response of the SSHSC models to be calculated.

The electrical and thermal properties used in this study are listed in Table 1. Each material of the structures was assumed to be continuous, homogeneous, and isotropic. The electrical conductivity of the silicon layers, adhesive layers, and silicon substrate was specified to be representative of highly resistive materials; thus, a very low number was used. The electrical conductivity of the polysilicon resistor was set to be 2404.5 (S/m) such that the electric current flowing through the copper meander in the FE model is equivalent to the measured current values, which are 6 mA, 8 mA, and 10 mA at 3 V, 4 V, and 5 V, respectively. In addition, we assumed that the Joule heat fraction, which is used to specify the fraction of dissipated electrical energy released as heat, is equal to one. The physical and thermal properties of the silicon layer and adhesive layer were identical to the values used for calculating the thermal diffusion length ($\mu_{eff}$). For the SSHSC and TSV models, fine FE meshes of 346,237 nodes and 313,824 hexahedral elements, and 321,039 nodes and 294,936 hexahedral elements, respectively, were used. Thus, the calculated results are considered to be insensitive to further refinement or increase in the number of elements.

### 4.2. Boundary Conditions and Finite Element Analysis

The procedure of the LIT process presented in the experiments of this study was mimicked by imposing suitable electrical and thermal initial conditions as described in Equation (16). The initial conditions for the electric potential and temperature of whole bodies are:

(16) $$\begin{array}{c} {V|}_{t=0}=V_{0}=0\;V\\ {T|}_{t=0}=T_{0}=293.15\;K \end{array}$$

For all outer surfaces except the bottom surface of the silicon substrate, a Robin boundary was applied to be the convective condition:

(17) $$-k\frac{\partial T(r,t)}{\partial n}=h(T_{s}-T_{\infty })$$

where h is the convection coefficient (W/m^2^·K), and $T_{s}$ and $T_{\infty }$ represent the temperature at the body surface and ambient temperature of the environment, respectively. Considering the normal LIT test conditions for laboratory operation, the convective condition was assumed to be the natural convection condition at room temperature, for which the values of h and $T_{\infty }$ are 3 W/m^2^·K and 293.15 K, respectively. A sinusoidal modulated bias voltage was applied to the ends of the probe region, whereas the ground condition was applied to the opposite side of the probe region:

(18) $$\begin{array}{c} V\;=\;V_{in}+V_{in}\sin(2\pi f_{lock-in}t)\\ V\;=\;0 \end{array}$$

The applied bias voltages (V) were 3 V, 4 V, and 5 V, with various lock-in frequencies (1, 2, 3, 4, and 5 Hz for the two-layer SSHSC model, and 2, 4, 6, 8, and 10 Hz for the three-layer SSHSC model and TSV model). In order to compare the phase shift (*ϕ*) of the resistive heating experiment and simulation, the coupled electro-thermal simulations were run for five cycles at each lock-in frequency, which is in thermally transient state. Transient state calculations based on the FE method were carried out using the commercial software ABAQUS (v6.8.3, Dassault Systèmes, Vélizy-Villacoublay, France).

## 5. Results and Discussion

**Figure 6:**
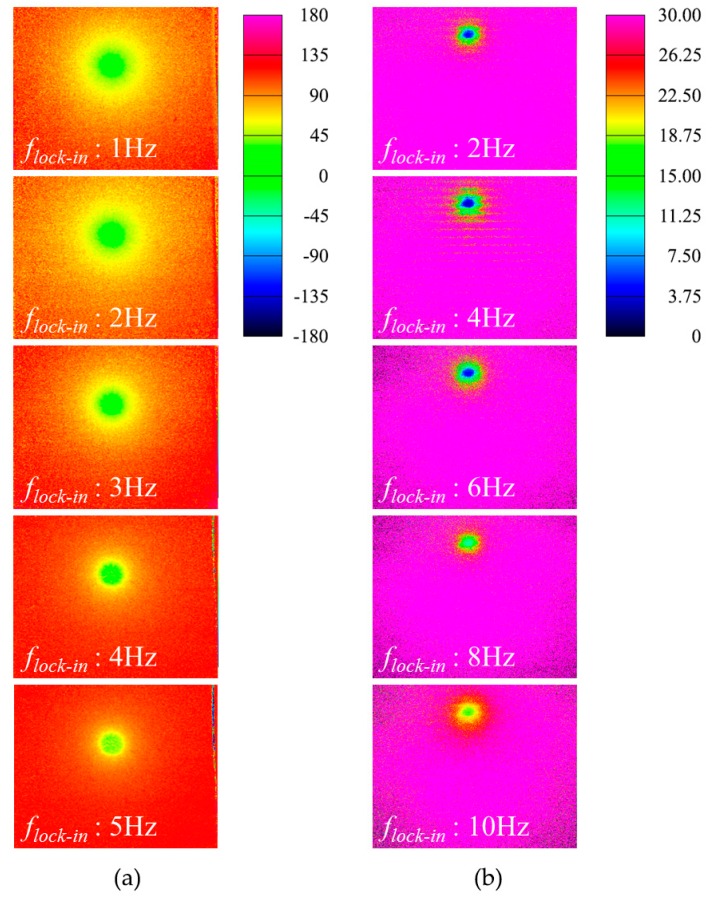
Phase images of SSHSCs for various lock-in frequencies with 4 voltage bias input: (**a**) two-layer SSHSC; (**b**) three-layer SSHSC.

The lock-in technique was used to reconstruct the phase image of registered thermal sequences, and the resultant phase images were evaluated for different lock-in frequency and bias voltage. Figure 6a,b show the representative phase images of the two SSHSCs for various lock-in frequencies with bias voltage of 4 V. The halo in Figure 6 indicates the heat generation of the polysilicon resistor that exists at the bottom of the SSHSC; therefore, an exothermic fault, which was not clearly detected by conventional infrared imaging techniques such as passive infrared imaging and pulse thermography, was more clearly isolated by lock-in operation [23,24]. In addition, the contrast created by the halo in the phase image has increased for increasing lock-in frequency. Basically, because a lock-in operation of high frequency utilizes more periods during calculation, it naturally leads to the calculation of a high-contrast phase image with high frequency. This phenomenon was well verified by the phase images of Figure 6, which are calculated for increasing lock-in frequencies.

**Figure 7:**
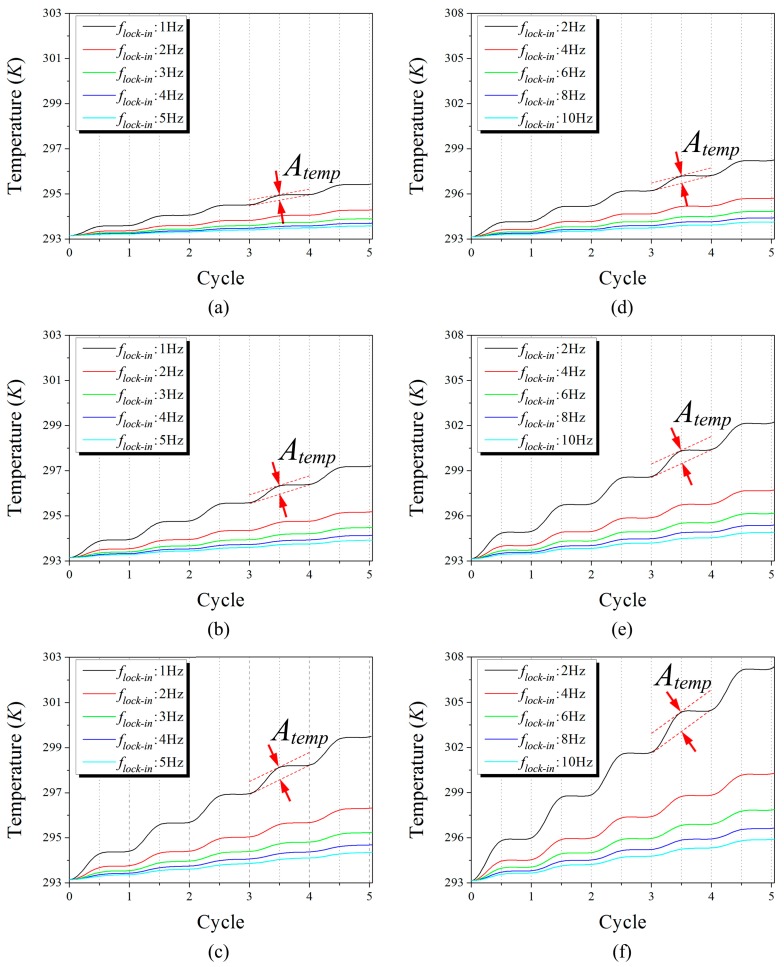
Numerical simulation results on the transient thermal responses for the whole range of lock-in frequencies with different bias voltages, by representing the continuous cycle: (**a**) 3 V biased two-layer SSHSC; (**b**) 4 V biased two-layer SSHSC; (**c**) 5 V biased two-layer SSHSC; (**d**) 3 V biased three-layer SSHSC; (**e**) 4 V biased three-layer SSHSC; (**f**) 5 V biased three-layer SSHSC.

An analysis of the thermal response obtained from the numerical simulation showed that the thermal diffusion mechanism in the multi-layered structure was clearly represented, and the phase shift was analyzed using lock-in method. Figure 7 shows the numerical simulation results on the transient thermal responses for the two-layer and three-layer SSHSC for the whole range of lock-in frequencies with different bias voltages, by representing the continuous cycle. The temperature was measured at a point located on the surface at a vertical distance from the heat source (see Figure 5a). For all cases, the amplitude of the periodical temperature ($A_{temp}$) was raised with an increase in the bias voltage, whereas the size of $A_{temp}$ diminished with an increase in the lock-in frequency at the same voltage. A comparison of the two-layer SSHSC (thick) with the three-layer SSHSC (thin) under the same Joule heating conditions revealed that $A_{temp}$ was larger for the thin specimen than the thick one. From these results, we could suppose that $A_{temp}$ is basically related with the magnitude of the $P_{loss}$ (according to the electric power formula $P_{loss}=V^{2}/R$), the heating time within one cycle is determined by the lock-in frequency, material thickness, and configuration.

**Figure 8:**
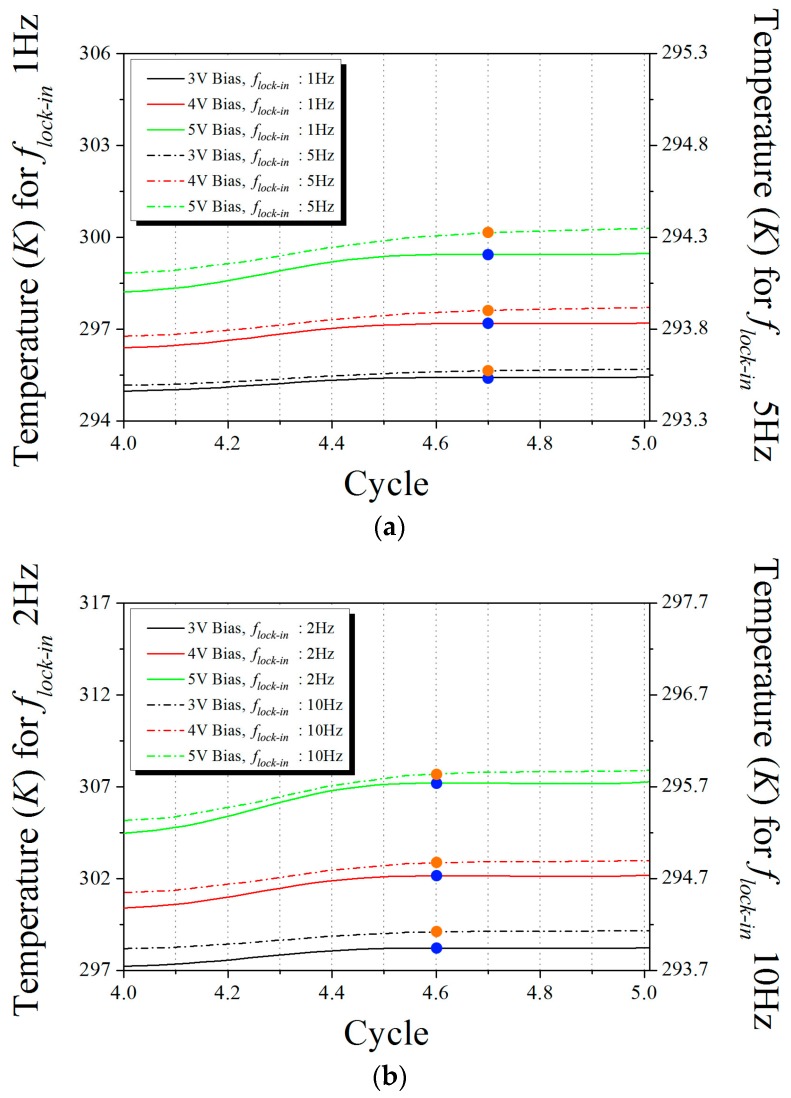
Enlarged view of the thermal response profiles for all bias voltages with two lock-in frequencies: (**a**) 1 Hz and 5 Hz for the two-layer SSHSC; (**b**) 2 Hz and 10 Hz for the three-layer SSHSC, at final cycle.

For a detailed analysis, we enlarged the view of the final cycle in Figure 7 of the thermal response profiles for all bias voltages with lock-in frequency of 1 and 5 Hz for the two-layer SSHSC and 2 and 10 Hz for the three-layer SSHSC, respectively, as shown in Figure 8. As can be found by the marked orange and blue point indicating the peak temperature in a cycle, the locations within the time period were almost identical regardless of the fact that $A_{temp}$ depends on the magnitude of $P_{loss}$ and the lock-in frequency. In other words, even though the size of $A_{temp}$ was varied according to the aforementioned factors, only the material thickness and configuration can affect the phase shift. Consequently, these phenomena imply that the control of $A_{temp}$ with the aim of stably detecting the thermal signal by the infrared camera would be unaffected when obtaining the real phase shift of the multi-layered structure during experimental LIT operation.

**Figure 9:**
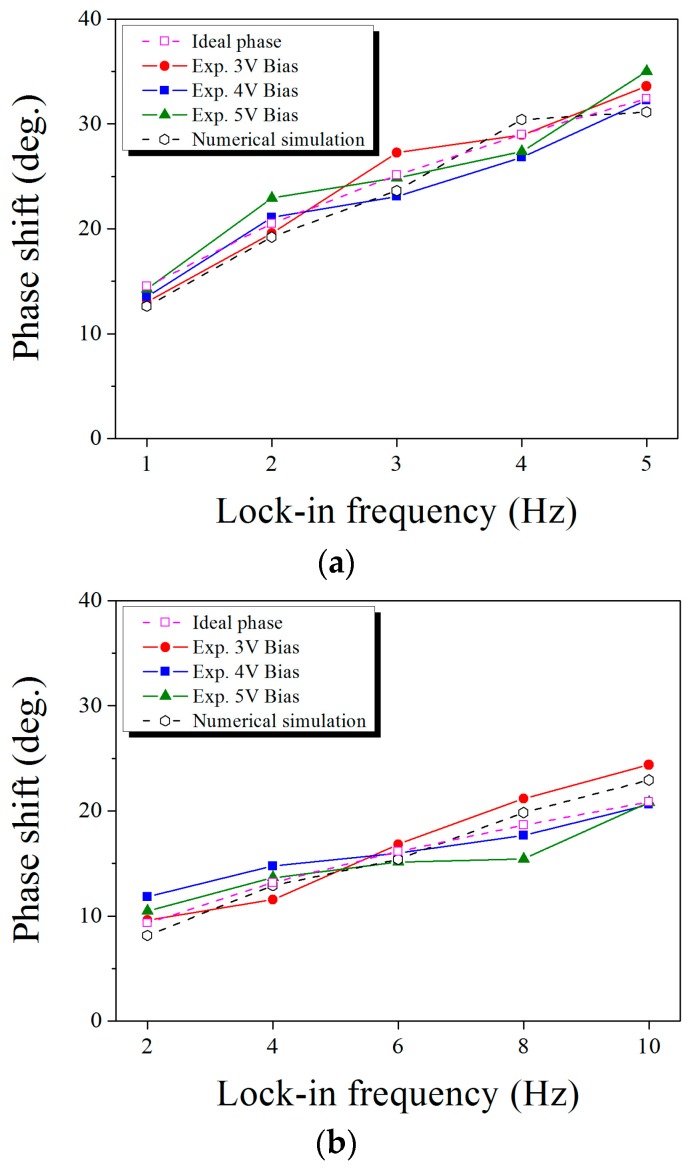
Phase shift of SSHSCs for different lock-in frequency and bias voltage: (**a**) two-layer SSHSC; (**b**) three-layer SSHSC.

Figure 9 presents the phase values that were estimated by both experiments and FE simulations for various lock-in frequencies and bias input voltages. The ideal phases were calculated by Equation (10) by considering both the actual depth of heat source buried in specimen and the physical configurations of each stacked material. The experimental phases were analyzed by employing the full width at half-maximum (FWHM) value of the respective phase image in Figure 6. The phase average of the circular area, which was decided by the FWHM of its phase image, determined the experimental phase, and numerical phases were calculated based on the transient thermal response profiles as presented in Figure 7. The phase shift results of numerical simulation shown in Figure 9 are calculated for 4 V of the bias voltage because the phase shifts were the same for different bias input voltages. As shown in Figure 9, the phase shifts of the experiment and numerical simulation simultaneously increased as the lock-in frequency increased.

In case of two-layer SSHSC (Figure 9a), the averages of the experimental phase for the applied lock-in frequencies were estimated as 13.6, 21.2, 25.07, 27.7, and 33.62 degrees, respectively, and the percent deviation of each numerical phase for the corresponding lock-in frequency was evaluated as 7.11%, 9.35%, 5.68%, 9.83%, and 7.36%, respectively. The difference between the ideal phase and experimental phase average for the respective lock-in frequency was estimated as 0.9, 0.69, 0.05, 1.29, and 1.19 degrees, respectively, on the basis of the ideal phase. Furthermore, the difference between the ideal phase and numerical phase average for the respective lock-in frequency was estimated as 1.87, 1.29, 1.47, 1.43, and 1.28 degrees, respectively, on the basis of the ideal phase.

In case of the three-layer SSHSC (Figure 9b), the experimental phase averages were estimated as 10.63, 13.32, 15.99, 18.08, and 21.94, respectively, for the corresponding lock-in frequency, and the percent deviation with each numerical phase for the corresponding lock-in frequency was evaluated as 23.41%, 3.15%, 3.72%, 9.75%, and 4.59%, respectively. The difference between the ideal phase and experimental phase average for the respective lock-in frequency was estimated to be 1.31, 0.13, 0.16, 0.56, and 1.09 degrees, respectively, on the basis of the ideal phase. In addition, the difference between the ideal phase and numerical phase average for the respective lock-in frequency was estimated as 1.18, 0.29, 0.76, 1.2, and 2.1 degrees, respectively, on the basis of the ideal phase.

Following the relationship between the phase and depth described by Equation (10), the depth of the heat source buried within each SSHSC was estimated by the experimentally and numerically acquired phase shift results provided in Table 2 at different lock-in frequencies. In the case of the two-layer SSHSC with an actual depth of 1342 μm, the depth for the experimental averages was estimated as 1258.63, 1387.28, 1339.52, 1282.08, and 1391.43 μm, respectively, and the depth for the numerical averages was calculated as 1169.11, 1257.51, 1263.38, 1408.12, and 1289.06 μm, respectively, for the applied lock-in frequencies. The difference between the actual depth and experimental depth average for the respective lock-in frequency was estimated as 83.37, 45.28, 2.48, 59.92, and 49.43 μm, respectively, which corresponds to 6.21%, 3.37%, 0.18%, 4.46%, and 3.68% on the basis of the actual depth. Further, the difference between the actual depth and numerical depth average for the respective lock-in frequency was estimated as 172.89, 84.49, 78.62, 66.12, and 52.94 μm, respectively, which corresponds to 12.88%, 6.3%, 5.86%, 4.93%, and 3.94% on the basis of the actual depth.

In case of the three-layer SSHSC with an actual depth of 170 μm, the depth for the experimental averages was estimated as 193.88, 171.70, 168.28, 164.87, and 178.88 μm and the depth for the numerical averages was calculated as 148.50, 166.29, 161.98, 180.96, and 187.09 μm, respectively, for the applied lock-in frequencies. The difference between the actual depth and experimental depth average for the respective lock-in frequency was estimated as 23.88, 1.70, 1.72, 5.13, and 8.88 μm, respectively, which corresponds to 14.05%, 1.01%, 1.01%, 3.02%, and 5.22% on the basis of the actual depth. Further, the difference between the actual depth and numerical depth average for the respective lock-in frequency was estimated as 21.50, 3.71, 8.02, 10.96, and 17.09 μm, respectively, which corresponds to 12.64%, 2.17%, 4.69%, 6.44%, and 10.05% on the basis of the actual depth.

In the depth estimation of the heat source in the two-layer SSHSC, deviations of 0.18% (2.48 μm) and 3.94% (52.94 μm) were the best values obtained for the experimental and numerical simulation comparison with the actual depth, respectively, and the deviations of 1.01% (1.70 μm) and 2.17% (3.71 μm) were the best for experimental and numerical simulation in comparison with the actual depth, respectively, in the application of three-layer SSHSC. These statistics summarized the results of both the experiments and the numerical simulation for the two different target specimens, and the feasibility of this experimental and numerical approach for depth estimation was well demonstrated.

**Figure 10:**
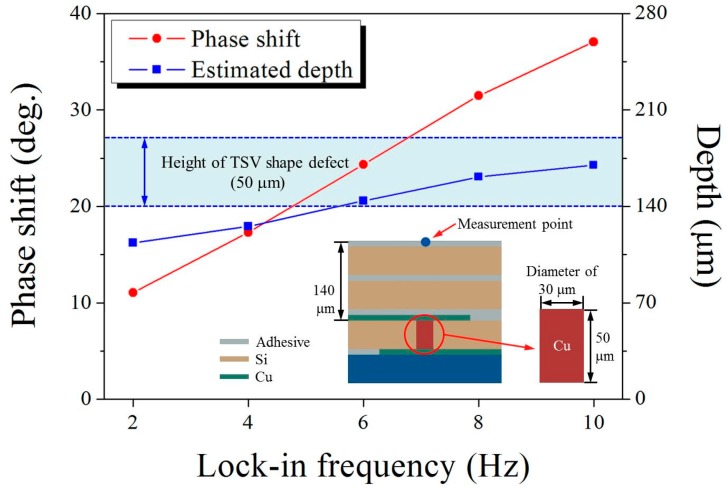
Phase shift and estimated depth of the 3D TSV heating source for lock-in frequencies with 3 voltage bias input.

The numerical simulation was additionally carried out to investigate the effects of the volumetric heat source configuration on the depth estimation, mimicking the 3D TSV integration package. The TSV volumetric heat source was assumed to be a cylindrical shape with a diameter of 30 µm and height of 50 µm, and was buried under adhesive, silicon, and copper layers with a total thickness of 140 µm, as shown in Figure 10. The phase shift and depth with different lock-in frequencies were assessed by following the numerical approach used in this study, and are presented in Figure 10 with the height of the buried TSV heat source. As expected, the values of the calculated phase shift increased with an increase in the lock-in frequency. However, the estimated depth at lock-in frequencies of 2 and 4 Hz fell short of the location of the TSV heat source, whereas this value at the higher frequencies of 6, 8, and 10 Hz of the lock-in frequency was within range of the TSV height. Similar to what we found in the results of the three-layer SSHSC (thin), the estimated depth at the lower lock-in frequency has a relatively larger error compared to that at the higher lock-in frequency. This trend coincided well with the results of Kijkanjanapaiboon et al. [19], who determined that, when a specimen becomes thinner at a lower lock-in frequency, the difference in its phase shifts becomes significant.

## 6. Conclusions

Construction and experiments of an infrared microscopy system, and FE simulation of 3D models were performed by using lock-in thermography technique through experimental and numerical approaches for the depth estimation of heat source buried in multi-layered silicon wafer architecture. Two test samples (silicon wafer stacked heat source chips) were fabricated and 3D models of them were developed for the LIT experiments and FE simulation, and showed reasonable performance for the depth estimation of heat source buried in multi-layered IC samples. In case of a two-layer SSHSC with an actual depth of 1342 μm, 0.18% (2.48 μm) and 3.94% (52.94 μm) were the best deviations between experimental test and numerical simulation comparison with the actual depth, respectively, and the deviations of 1.01% (1.70 μm) and 2.17% (3.71 μm) were the best for experimental test and numerical simulation in comparison with the actual depth, respectively, for the three-layer SSHSC with an actual depth of 170 μm. Effects of the volumetric heat source configuration on the depth estimation was numerically analyzed by modelling TSV mimicking volumetric heat source, and results showed satisfactory performance of depth ranged from 140 μm to 190 μm. These findings demonstrate that our approach of lock-in thermography technique using an infrared microscopy and FE simulations for the depth estimation of heat source can be potentially useful as the nondestructive testing (NDT) methodology for 3D defect localization in fully packaged devices. Even though our results are promising, the limitations of the lock-in method are still remains and further studies are needed to develop more robust infrared microscopy system that can resolve the limitations of spatial and temperature resolution, and any fault dependence of the 3D geometry that may exists in a packaged device.

## Figures and Tables

**Table 1:** Electrical and thermal properties used for the calculation of the effective thermal diffusion length ($\mu_{eff}$).

Thermo-Physical Properties of Each Layer					
	Silicon Layer	Adhesive Layer	Poly-Silicon Resistor	Cu Meander	Silicon Substrate
Density, ρ, (kg/m^3^)	2329	1250	2320	8960	2329
Specific heat capacity, $C_{p}$, (J/kg·K)	710	1110	678	384	700
Thermal conductivity, k, (W/m·K)	149	0.14	34	401	130
Electric conductivity, σ, (S/m)	1 × 10^−12^	1.776 × 10^−13^	2.4045 × 10^3^	5.81 × 10^7^	1 × 10^−12^
Dielectric constant	1	8.32	4.5	1	1

**Table 2:** Estimated depth for the two-layer SSHSC and three-layer SSHSC.

**Depth Estimation for Two-Layer SSHSC (µm)**					
**Lock-In Frequency (*f_lock-in_*)**	**Experiment**			**Numerical Simulation**	**Actual Depth**
	**3 V**	**4 V**	**5 V**		
1	1203.25	1252.77	1319.87	1169.12	1342
2	1280.82	1380.63	1500.40	1257.51	
3	1456.73	1233.09	1328.74	1263.36	
4	1338.62	1240.74	1266.88	1408.15	
5	1390.19	1335.55	1448.55	1289.02	
**Depth Estimation for Three-Layer SSHSC (µm)**					
**Lock-In Frequency (*f_lock-in_*)**	**Experiment**			**Numerical Simulation**	**Actual Depth**
	**3 V**	**4 V**	**5 V**		
2	175.01	215.85	190.77	148.50	170
4	149.06	190.13	175.92	166.29	
6	177.09	168.36	159.40	161.98	
8	192.96	161.07	140.57	180.96	
10	198.83	168.07	169.73	187.09	
